# Hybrid reference genome assembly for the archetypal *Leishmania (Viannia) guyanensis* bearing the dsRNA totivirus LRV1

**DOI:** 10.1128/mra.00992-22

**Published:** 2025-07-22

**Authors:** Peter J. Myler, Gowthaman Ramasamy, Wesley C. Warren, Deborah E. Dobson, Stephen M. Beverley

**Affiliations:** 1 Center for Global Infectious Disease Research, Seattle Children’s Research Institute, Seattle, Washington, USA; 2 Department of Pediatrics, University of Washington 271845, Seattle, Washington, USA; 3 Department of Biomedical Informatics and Medical Education, University of Washington 312757 , Seattle, Washington, USA; 4 Department of Animal Sciences, Institute of Data Science and Informatics, Bond Life Sciences Center, University of Missouri 14716, Columbia, Missouri, USA; 5 Department of Molecular Microbiology, Washington University School of Medicine, St. Louis, Missouri, USA; Loyola University Chicago, Chicago, Illinois, USA

**Keywords:** *Leishmania guyanensis*, *Viannia*, comparative genomics, mucocutaneous leishmaniasis, kinetoplastida, Trypanosomatidae, South America

## Abstract

We report a high-quality hybrid genome assembly and annotation for a *Leishmania (Viannia*) *guyanensis* line (M4147) expressing a firefly luciferase reporter gene and bearing the totivirus LRV1 strongly implicated in parasite hypervirulence. This reference genome should facilitate the study of infectivity and pathogenesis of cutaneous and severe leishmaniasis.

## ANNOUNCEMENT

The trypanosomatid parasite *Leishmania (Viannia) guyanensis* is transmitted by phlebotomine sand flies and causes American tegumentary leishmaniasis (ATL). These infections are primarily zoonotic, but cause a range of human diseases, from localized or disseminated cutaneous lesions to disfiguring mucocutaneous disease (1), with both parasite and host factors contributing to different clinical manifestations. We report a hybrid short- and long-read assembly of a WHO reference line bearing the archetypal protozoal dsRNA totivirus LRV1 (2, 3), which has been used extensively since 1991 for studies of LRV1 biology and RNA interference (4–6). The clonal line (LgyM4147-LUC3) sequenced here expresses high levels of firefly luciferase, facilitating *in vivo* bioluminescent analysis, including LRV1-driven metastasis (5–7). Previous assemblies of *L. guyanensis* using short-read (Illumina) data contained numerous sequence gaps (8); but the present hybrid assembly comprises 35 chromosomal contigs (with only 4 sequence gaps) and the mitochondrial kinetoplast maxicircle. The LRV1 dsRNA virus sequence was also revised (see Table 1).

**TABLE 1:** Summary of *Leishmania (Viannia)* guyanensis hybrid assembly

Parameter	Value
*L. guyanensis* strain	LgyM4147Luc3
WHO code	MHOM/BR/75/M4147
Source	Human, cutaneous
Provenance	Jean Patterson S.M. Beverley
Bioproject	PRJNA872130
Assembly accession number	GCA_024970365.1
Read data accession numbers	Illumina: SRR21440856 PacBio SRR21440857
HGAP3 assembly	167 unitigs N_50_ = 712 kb
Chromosomes	35 + Maxicircle
GC content	57.9%
Assembly size	32,544,702 bp
Protein-coding genes	8,066 CDSs + 149 pseudogenes
RNA genes	25 rRNAs, 77 tRNAs, 1,077 ncRNAs
Transposable elements	53 TATEs + 14 SLACs
LRV1 accession number	KX808487.1

Parasites were cultivated in Schneider’s medium at 26°C in sealed flasks (5), grown to late log phase (3–4 days), and harvested by centrifugation. Nuclei were purified (9) and extracted by two rounds of gentle phenol/chloroform extraction (10), yielding gDNA >20 kb. DNA was sheared using a Covaris instrument and size-selected (>12 kb) with the Blue Pippen system (Sage Science) to generate libraries using the SMRT (single molecule real time) bell template kit 1.0, according to the manufacturer’s protocol (Pacific Biosciences). These were sequenced on an RSII instrument (Pacific Biosciences) using P6-C4 sequencing chemistry with a “movie length” of 4 h on a total of four SMRT cells, producing a total of 654 k reads with a mean and N50 read lengths of 4,827 and 11,634 bp, respectively.

Unless otherwise stated, version 1 and default parameters were used for all software tools. After quality filtering SMRT sequences to 26.7× estimated coverage, *de novo* assembly was performed with HGAP version 3 (11). Assembled contigs were error-corrected using Quiver (12) to generate high-quality consensus sequences. 13.5M (100 bp paired-end) reads generated on an Illumina HiSeq2000 instrument previously (8) were quality-filtered using QIIME (13) and used to further polish the Quiver-corrected contigs by 10 iterations of ICORN v2 (14). The resultant 167 unitigs were scaffolded by alignment against the *L. braziliensis* M2904 genome (15) using ABACAS v2 (16), resulting in 35 pseudochromosomes and one maxicircle contig. Gaps (and missing ends) were filled using PBJelly v2 (17) followed by manual curation, yielding a final genome length of 32,544,702 bp, with only 4 gaps and 31 of the 70 chromosome ends reaching telomere-associated hexanucleotide sequences (15).

Automated annotation was subsequently performed using RATT (18), SNAP (19), Augustus (20), Aragorn v1.2.38 (21), and Infernal v1.1 (22), followed by manual annotation with BlastN (23) to identify transposable elements (TEs) (24). The number of protein-coding and RNA genes (Table 1) was similar to those found in other *Leishmania* genomes (15, 25).

## Data Availability

Sequence reads and assembly data were deposited in the NCBI GenBank repository under BioProject PRJNA872130, with accession numbers and hyperlinks found in Table 1.
